# Description of a Remarkable Enlargement of the Ovaria

**Published:** 1802-11-01

**Authors:** H. Vanden Bosch

**Affiliations:** Wageningen


### *Description of a remarkable Enlargement of the Ovaria; communicated by* H. VANDEN BOSCH, M.D. *of Wageningen.*

IN the month of July 1797, I was deſired to attend D. Janſen, a poor woman, who was ſuppoſed to be dropſical. She appeared extremely emaciated. Her abdomen was amazingly large, hanging down to her knees, and meaſuring rather more than four feet eight inches in circumference. She complained of great weight and pain in the lower part of the abdomen and backwards juſt over the right hip. Upon enquiring into her hiſtory that I might aſcertain the cauſe of this ſingular appearance, I learned that about five years ago, while ſhe was with her huſband, who was a ſoldier in garriſon, ſhe perceived a tightneſs in the lower belly, and a hardneſs which gradually increaſed; ſo that upon removing to town, ſhe conſulted ſome females, who pronounced her to be with child; the midwife not only confirmed their opinions, but aſſured her that ſhe would have twins. But theſe aſſurances proved fallacious.

In the ſevere winter 1794, 1795, ſhe was compelled to remove to Weeſop, where ſhe was ſeized with a violent fever, which was accompanied with an alarming *hæmorrhagia uteri*; but upon her recovery, the abdomen began to enlarge very conſiderably. Her anxiety increaſing, ſhe conſulted the faculty at every place where ſhe came; but with no ſucceſs. Nor had the *paracentesis abdominis*, performed in the year 1796, by the Surgeon Major of the regiment, any better effect than the medicines ſhe had uſed. Not more than two cups full of a gelatinous fluid were diſcharged from two openings that were made.

Notwithſtanding the greatneſs of the diſtention, the uterus was was not enlarged. She was ſeldom feveriſh; nor when ſhe ſat ſtill and compoſed, was reſpiration difficult. Motion became came gradually more troublesome to her, till at last it was impracticable. She had her monthly courses regularly; there were seldom indications of a *flour albus:* The urine was often discharged with considerable pain, and it had a lateritious sediment. In short, all the natural functions were much less impeded, even to the day of her death, than might have been expected.

All the above symptoms, united with the ill success of the *paracentesis*, induced me to suspect that the distention proceeded from a tumour, degeneration, or dropsy in the *ovarium*; and, accordingly, I had recourse to palliative medicines. The distention increasing, I wished her to submit to a second operation, under the idea that extreme pressure might occasion a kind of symptomatic dropsy; but as I could not *promise* success she refused to comply. A self-sufficient surgeon in the town, who assured her of a cure, and abused me for trifling with a complaint that was so easily remedied, obtained her consent. He undertook the operation in January 1798, and after three openings could not draw off more than about a quart of fluid; he tried various medicines, but in vain. Of consequence she lost her confidence in him, and my assistance was again requested. I did my utmost to alleviate her misery, till the month of July 1800, when she expired.

I was prevented by severe indisposition from opening the body; but foreseeing the event, I had desired my friend Mr. F. H. Hartog, a medical pupil in the University at Utrecht, to perform the operation, and communicate to me whatever appeared worthy of notice. My friend performed this office with skill and attention, and transmitted to me the following important discoveries, which fully justified my conjectures. I shall relate the particulars in his own words, premising only that the envelope of the abdomen, which had long been supported by a suspensorium, measured in the month of July 1800, somewhat more than two ells and a half in length.

" Upon placing the body on the table, the abdomen appeared of an enormous size. It was about five feet and a half in circumference. It was hard to the touch; and various lumps or knobs were easily distinguishable. The navel was of the size of my fist. On the right side, the belly was more protuberant than on the other. Upon making an incision through the skin, we observed that the celular membrane between the cutis and the muscular parts on the left side, was very thick, and seemed a kind of saponacious substance; but on the right side there were scarcely any traces of this membrane. The muscles on the left side were of the natural size; on the other they were thin and emaciated. Upon opening the cavity, about sixteen pints pints of flimey and offenfive matter iffued out from the parts juft above the umbilical region.

" The inteftines were moftly in the upper part of the abdomen. A large, irregular, and preternatural fubftance occupied almoft the whole of the remainder. This was taken out, being feparated from its union with the *vagina*, *uterus*, &c. that we might more minutely examine the ftate of the body as well as the fubftance itfelf. The liver was in a natural ftate. The bile in the gall bladder was thicker and of a deeper colour than is ufual. The *spleen*, *pancreas*, and *kidneys*, retained their form and confiftency; the *ureters* were, at their origin, more expanded than is natural. The fmaller inteftines were not injured; the larger, particularly the *colon transversum*, were diftended with fæces and air: the larger veffels were as ufual. The lungs were remarkably red, but appeared in a found ftate; as alfo the heart, though it was replete with blood, which poured in large quantities into the cavity of the thorax, when it was opened.

" The misformed fubftance had a red flefhy appearance; it was covered with a very thin membrane. On the right fide it was prominent, and was fomewhat excavated on the left. It feemed to be compofed of feveral pieces of a fub-globular form, which were feparated from each other by fiffures or grooves, from four to five inches in depth, but adhering to each other at their bafis. They refembled the gyræ obfervable in the cortical fubftance of the brain. This fubftance was eighteen inches in length, and about nineteen inches in diameter. In the middle of this body, rather towards the right fide, was a *saculum*, formed of a thick membrane. This cavity meafured feven inches from beneath upwards, and five in depth; but it had no communications. It contained nearly five pints of mucilaginous matter. It was furrounded by the fub-globular bodies mentioned above, which upon mature examination, feemed to confift of a hard flefhy fubftance; and in fome places a fubftance refembling the *materia adiposa* was perceived. One thing obfervable in this fac was, that its internal furface was not fmooth and even, but had various irregular filaments running acrofs it, in different directions. Towards the lower part of the cavity, but neareft to the upper furface of the body, three fmall cavities were obfervable; thefe contained a much thicker fluid than the other part. The *ureters* ran in grooves behind this fubftance; their opening into the *vesica urinaria* was natural. This vifcus being ftrongly attached to the inferior part of the fubftance was much compreffed, contained very little urine, but feveral red particles, with which the *urethra* was alfo filled.

" The *uterus* was more oblong than ufual, and inclined towards wards the left ſide, as did alſo the *vagina*; but they appeared in a ſound ſtate.

"The right *Fallopian tube* was open from its origin to the diſtance of about three lines from the *uterus*, where it became obſtructed, and ſtrongly adhered to the preternatural ſubſtance. Every trace of it was now loſt, nor could a natural *ovarium* be perceived. The left Fallopian tube was perfect, and open through its whole length; the *fimbriæ* alſo were eaſily diſtinguiſhed. This tube, however, was firmly attached to the cartilaginous part of the large body. This cartilaginous maſs was alſo compoſed of ſeveral large knobs; and ſome of them were about four times the ſize of my fiſt. They adhered ſo firmly at their baſis, that they were ſeparated with difficulty. No ovarium could be diſcovered on this ſide alſo.

"The weight of this great misſhapen body was not leſs than one hundred and two pounds. As there was no appearance of *ovaria*, either on the right or left ſide; and as the tubes on each ſide were incorporated with this ſubſtance, I do not heſitate to pronounce that this was a *degeneratio ovariorum*; and that the *Ovaria* adhering and growing together, formed themſelves into this enormous maſs."

